# *FOXP4* Variants Are Associated With Plateau Iris and Angle Closure Glaucoma

**DOI:** 10.1167/iovs.66.9.23

**Published:** 2025-07-10

**Authors:** William Presley, Su Qing Wang, Bin Guan, Seong Hoon Jeong, Chelsea Bender, Erika Ward, Kayla Johnson, Bridget Blevins, Natalie Michaels, Manjool Shah, Sayoko E. Moroi, Robert B. Hufnagel, Julia E. Richards, Lev Prasov

**Affiliations:** 1 Department of Human Genetics, University of Michigan, Ann Arbor, Michigan, United States; 2 Department of Ophthalmology and Visual Sciences, University of Michigan, Ann Arbor, Michigan, United States; 3 Ophthalmic Genetics and Visual Function Branch, National Eye Institute, National Institutes of Health, Bethesda, Maryland, United States; 4 Department of Ophthalmology and Visual Sciences, The Ohio State University School of Medicine, Columbus, Ohio, United States; 5 Center for Integrated Healthcare Research, Kaiser Permanente Hawaii, Honolulu, Hawaii, United States; 6 Department of Epidemiology, University of Michigan, Ann Arbor, Michigan, United States

**Keywords:** plateau iris, angle closure glaucoma, FOXP4, anterior segment dysgenesis, forkhead transcription factor

## Abstract

**Purpose:**

Angle closure glaucoma (ACG) is a common cause of adult-onset vision loss that often presents with iris abnormalities and short axial lengths. Although it is heritable, little is known about the genetic risk factors underlying this condition. We thus conducted a disease gene discovery study in a family exhibiting an autosomal dominant triad of ACG, plateau iris, and short axial lengths.

**Methods:**

Pooled exome sequencing was performed to identify coding variants contributing to disease. The spatiotemporal expression pattern of candidate gene *FOXP4* was evaluated via immunostaining in embryonic mouse eyes. YFP-tagged mutant and wild-type FOXP4 proteins were expressed in HEK-293T and ARPE-19 cells to evaluate nuclear localization, and an SRPX2-Luciferase reporter was used to ascertain variant effects on transcriptional regulation. We also reviewed more than 20,000 patients (primarily from the UK Biobank) diagnosed with glaucoma and/or disorders of the iris and ciliary body for additional *FOXP4* variants and functionally validated them as described.

**Results:**

We identified a single likely pathogenic variant in transcription factor *FOXP4*: c.1433A>G (p.Q478R). *FOXP4* is highly expressed in multiple structures relevant to the drainage angle, such as the periocular mesenchyme, iris, ciliary body, and cornea. The p.Q478R variant appears to be a hypomorphic allele that retains its transcriptional activity, but often mislocalizes to cytosolic aggregates. Comparable variants, including one found in another glaucoma patient, show similar mislocalization that may indicate protein instability.

**Conclusions:**

These data suggest that *FOXP4* is important for anterior segment development and that variants therein are rare risk factors for ACG.

Glaucoma describes a group of ocular conditions characterized by the progressive degeneration of the optic nerve.^1^ It is a leading cause of adult-onset vision loss worldwide and often results in total blindness if left untreated.^1^^,^^2^ The different etiologies of this disease can be broadly categorized into two main subtypes: open angle glaucoma (OAG) and angle closure glaucoma (ACG). ACG confers a three-fold higher risk of irreversible blindness than OAG, despite accounting for only approximately one-third of total cases.^3^ ACG is often associated with hyperopia or short axial length and occurs when anatomical abnormalities in the anterior segment of the eye block the aqueous outflow tract.^4^ Such abnormalities include plateau iris, an anterior rotation of the ciliary body coupled with a flat iris configuration that can lead to both appositional acute and chronic angle closure.^5^ The resulting occlusion causes an increase in intraocular pressure, putting mechanical stress on the optic nerve and leading to optic atrophy.^4^ Unfortunately, many patients with chronic ACG still experience significant vision loss because they remain asymptomatic until later stages of the disease.^1^ Defining the genetic risk factors for ACG and associated anatomical defects may thus aid in better identifying and monitoring those at risk, although doing so has proven challenging despite the high heritability of this condition.^6^ This may be due, in part, to difficulties in assessing genomic segregation data in families displaying even Mendelian forms of adult-onset glaucoma. The high prevalence of glaucoma as an umbrella diagnosis—up to 7% of Caucasian individuals over the age of 65^7^—combined with the fact that this disease can be attributed to both complex genetic and nongenetic factors commonly results in instances of incomplete penetrance, phenocopy, and locus heterogeneity that can make pedigrees difficult to interpret.^8^^–^^11^

To date, *SPATA13*^10^ and *PCK2*^12^ remain the only putative Mendelian genes for ACG with significant functional evidence. A variety of other potential genetic risk loci have, however, been identified by linkage and genome-wide association studies (GWAS), molecular expression profiles, and animal models. Of particular note are genes involved with extracellular matrix formation and maintenance and cellular adhesion, as well as proposed regulators of axial length and anterior segment development.^6^ This includes transcription factors such as *ST18*, *CHX10*, *GLIS3*, and *MYRF*.^13^^–^^16^

Although forkhead domain transcription factors have not been studied previously in the context of plateau iris or ACG, they are intriguing candidates. These genes are important for anterior segment development and are associated with several anterior segment dysgenesis disorders (ASDs), as well as multiple glaucoma subtypes. Monoallelic stoploss mutations in *FOXE3* result in both mild anterior segment anomalies and ASDs, whereas biallelic missense and loss of function variants are present in patients with microphthalmia, glaucoma, and a variety of anterior segment findings.^17^ *FOXC1* variants, including deletions and duplications, have been reported in cases of ASD, Axenfeld–Rieger syndrome, and congenital or juvenile glaucoma.^18^ *FOXC1* is also a significant GWAS locus for adult-onset OAG, as are *FOXF1* and *FOXP4*.^19^

Here, we report on a family with autosomal dominantly inherited plateau iris and ACG, segregating together with short axial length. Using pooled exome sequencing, we identify a deleterious variant in the forkhead domain of *FOXP4* and provide additional genetic, functional, developmental, and in silico evidence supporting its role in ACG pathogenesis.

## Materials and Methods

### Human Subjects

Protocols were approved by the Institutional Review Board of the University of Michigan and the Office of Human Research Subject Protection at the National Institutes of Health per the Common Rule of the United States Federal Government (46CFR45). Participants provided written informed consent and were evaluated primarily at the University of Michigan Kellogg Eye Center or recruited remotely from other clinical sites with subsequent record review.

Many of the participants screened for *FOXP4* variants were recruited during previous genetic studies of short axial length/high hyperopia.^20^^,^^21^ Additional patients were added based on a diagnosis of plateau iris and/or ACG as assessed by gonioscopy or ultrasound biomicroscopy. Patients with gross ocular malformations were excluded.

### Pooled Whole-exome Sequencing and Variant Analysis

Pooled whole-exome sequencing was performed as previously described.^21^ Individual variant segregation was confirmed by PCR with the primers/conditions described in Supplementary Table S1, followed by Sanger sequencing of amplicons (Azenta Life Sciences, Waltham, MA, USA).

To rule out aberrant splicing in *FOXP4*, frozen blood samples from several family members carrying the variant were thawed in red blood cell lysis solution and RNA extracted using the Monarch Total RNA Miniprep Kit (New England Biolabs, Ipswich, MA, USA). Afterward, cDNA was prepared with the Superscript III One-Step RT-PCR System (Invitrogen, Waltham, MA, USA) and random hexamer primers according to the manufacturer's protocol. The intron-exon boundary near the *FOXP4* variant was then PCR amplified using the primers/conditions from Supplementary Table S1 and Sanger sequenced in both directions (Azenta Life Sciences, Waltham, MA, USA).

### Immunostaining and RNAscope in Mouse Eyes

Antibody staining was performed as previously described^21^ with a FOXP4 polyclonal antibody (ABE74, Sigma-Aldrich, Burlington, MA, USA) at a concentration of 1:350 and an Alexa Fluor 555 (red) conjugated secondary antibody. Nuclei were counter-stained with DAPI (MBD0015, Sigma-Aldrich) and mounted in ProLong Gold Antifade Mountant (P36930, Invitrogen).

RNAscope was performed with the Mm-Foxp4 probe (Advanced Cell Diagnostics, Newark, NJ, USA) and the RNAscope Multiplex Fluorescent Detect V2 system (Advanced Cell Diagnostics) per the manufacturer's protocol.

All tissue sections supplied for this study came from animals maintained in accordance with the ARVO Statement for the Use of Animals in Ophthalmic and Vision Research.

### Plasmid Generation

Previously validated YFP-tagged FOXP4 wild-type (WT) and p.H517N expression plasmids, as well as an SRPX2-Luciferase reporter and a pGL4.74 (TK) Renilla Luciferase construct, were obtained from Simon Fisher.^22^ YFP-tagged FOXP4 p.Q478R, p.V466I, p.V531I, p.R541Q, p.Q544P, and p.R546L were synthesized from the WT plasmid using the QuikChange XL Site-Direct Mutagenesis Kit (Agilent Technologies, Santa Clara, CA, USA) with the addition of 0.3–1x MasterAmp and the primers described in Supplementary Table S1. All constructs were subsequently validated by whole-plasmid sequencing (Plasmidsaurus, Eugene, OR, USA).

### Cell Culture and Transfections

HEK-293T cells were maintained at 37°C and 5% CO_2_ using DMEM with L-glutamine, 10% fetal bovine serum, and 1% penicillin-streptomycin (Gibco Life Technologies, Grand Island, NY, USA). All transfections were done with FuGENE (Promega, Madison, WI, USA) according to the manufacturer's instructions.

ARPE-19 cells were maintained under the same incubation conditions using F12/DMEM (1:1) media with 10% fetal bovine serum, 1% penicillin-streptomycin, and 1% nicotinamide (Gibco Life Technologies). All transfections were done with ViaFect (Promega) at a ratio of 1 µL per 6 µg of DNA, again according to the manufacturer's instructions.

### Luciferase Assays

We transfected 24-well plates seeded with 1.5 × 10^5^ HEK-293T cells/well at 70% confluency with 352.5 ng YFP-FOXP4 or pcDNA3.1 (-) empty vector, 117.5 ng of SRPX2-Luciferase, and 30 ng TK Renilla Luciferase. *Firefly*/*Renilla* luciferase ratios were then ascertained 24 hours later using the Dual-Luciferase Reporter Assay System (Promega) on a GloMax 96-well microplate luminometer (Promega) per the manufacturer's instructions and as previously described.^22^ To determine if significant differences between conditions could be observed at a lower transfection concentration, this assay was also repeated with 20 ng of YFP-tagged FOXP4, 100 ng of SPRX2-Luciferase, 10 ng TK Renilla Luciferase, and 370 ng of empty vector—either pcDNA3.1 (-) or pSPL3 (Invitrogen).

### HEK-293T Subcellular Localization Assays

Cells from the second round of luciferase assays (20 ng YFP-FOXP4/well) were visualized on an EVOS FLc inverted fluorescent microscope (Invitrogen). Approximately four nonoverlapping 20× images were taken by random sampling per well to ensure that mislocalization could be quantified manually across at least 100 cells per replicate in a blinded fashion.

To obtain representative images at 40× magnification, Nunc Lab Tek II Chamber Slides (Thermo Fisher Scientific, Waltham, MA, USA) were seeded with 5 × 10^4^ cells and transfected with 20 ng of YFP-FOXP4 and 480 ng of empty vector 18 to 24 hours later. After another 18 to 24 hours, cells were fixed in 4% paraformaldehyde for 10 minutes, permeabilized with 0.05% Triton-X in PBS for 5 minutes, and blocked with a solution of 1% BSA and 3% normal donkey serum in PBS for 1 hour at room temperature. Cells were next incubated at 4°C overnight with a 1:350 dilution of FOXP4 polyclonal primary antibody (ABE74, Sigma-Aldrich), washed, and stained with an Alexa Fluor 488 (green) conjugated secondary antibody for 2 hours and DAPI (MBD0015, Sigma-Aldrich) for 5 minutes at room temperature. Slides were imaged on a Leica DM6000 fluorescent microscope (Leica Microsystems Inc., Deerfield, IL, USA).

### ARPE-19 Subcellular Localization Assays

Six-well plates were seeded with 6 × 10^5^ cells and allowed to grow for 18 to 24 hours. The media was then aspirated and replaced with an antibiotic-free mixture of F12/DMEM (1:1), 10% fetal bovine serum, and 1% nicotinamide (Gibco Life Technologies) before cells were transfected with 100 ng of YFP-FOXP4. Cells were imaged on the EVOS FLc 48 hours later, as described.

### Patient Cohort Screenings

Individual whole-exome sequencing for ACG/plateau iris/high hyperopia patients was conducted as previously described,^20^^,^^21^ with samples/data processed in accordance with the protocols outlined. Additionally, UK Biobank Genebass^23^ patients classified under the following diagnostic codes were examined in the web browser (https://app.Genebass.org/) for variants in *FOXP4* (ENSG00000137166): “H40 Glaucoma,” “Glaucoma Custom,” “Glaucoma,” “Glaucoma Surgery/Trabeculectomy,” and “H21 Other Disorders of the Iris and Ciliary Body.” Variant enrichment statistics for each category were calculated automatically by the online interface.

### Transcriptomic, PROST, and Statistical Analyses

Pan-ocular gene expression of *FOXP1*, *FOXP2*, and *FOXP4* from a previously published single-cell RNA sequencing dataset^24^ was plotted on Spectacle.^25^ PROST analysis was conducted in accordance with the developer's instructions (https://github.com/ShahidIqb/PROST).^26^ Statistical significance for localization, luciferase, and stability assays was determined via one-way ANOVA coupled with Tukey testing.

## Results

### Pedigree and Molecular Analysis

**Figure 1.**
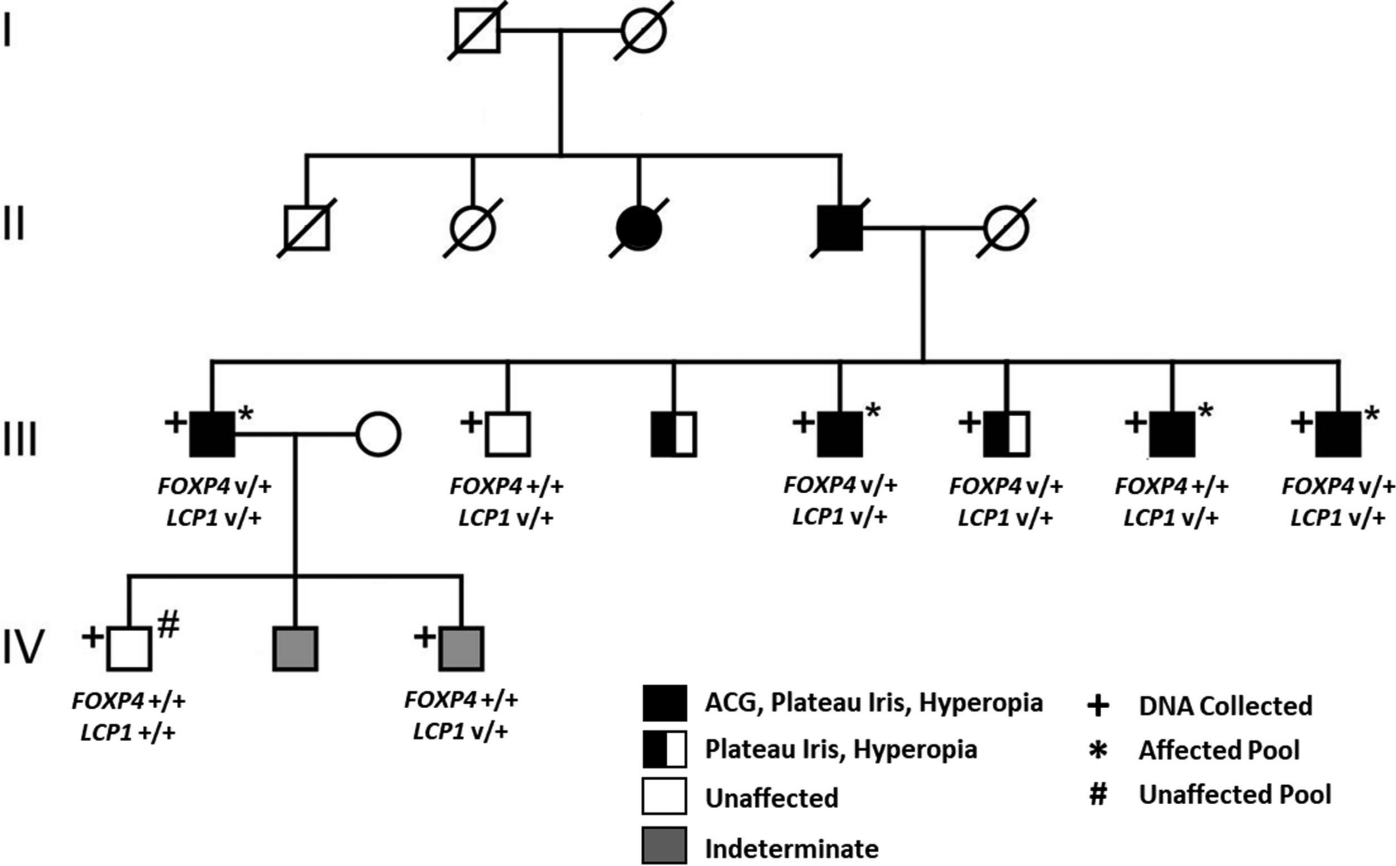
Pedigree showing the segregation of the *LCP1* (p.K534T) and *FOXP4* (p.Q478R) variants, along with disease status. II-3 and II-4 affected per family report.

We identified a four-generation pedigree of Ashkenazi Jewish descent presenting with a variable triad of plateau iris, adult-onset ACG, and short axial lengths inherited in an autosomal dominant manner (Fig. 1; Supplementary Table S2). Relevant branches include seven affected and two definitively unaffected family members. Four affected individuals from one generation were selected for pooled whole exome sequencing, and one definitively unaffected individual was sequenced separately as a control for variant exclusion. After ruling out mutations in known glaucoma-associated disease genes, we filtered for potentially deleterious variants found only in the affected pool and at an allele fraction consistent with an autosomal dominant disorder (0.3–0.7). We then prioritized any variant with a gnomAD^27^ (v2.1) total allele frequency of less than 5 × 10^−4^, which left no intronic variants. The remaining exonic variants were prioritized if they had a CADD^28^ score of greater than 20, a REVEL^29^ score of greater than 0.5, and were not predicted tolerated or benign by SIFT^30^ or PolyPhen.^31^ Two potential candidate variants met these pathogenicity criteria.

The first was missense substitution c.1601 A>C (p.K534T) in Lymphocyte Cytosolic Protein 1 (*LCP1*, ENST00000323076.7), a gene with ocular outflow tract expression that is limited to the vascular endothelial cells (Supplementary Fig. S1).^24^^,^^25^ However, this variant has a relatively high gnomAD v4.1 subpopulation allele frequency of 4 × 10^−3^ in Ashkenazi Jews and was found by Sanger sequencing to be present in all sampled siblings from the third generation of the pedigree regardless of plateau iris/glaucoma status (Fig. 1). It is therefore unlikely to be causal.

The other potential candidate was missense substitution c.1433 A>G (p.Q478R) in Forkhead Box P4 (*FOXP4*, ENST00000307972), which belongs to a family of transcription factors implicated in multiple forms of ASD and glaucoma.^17^^–^^19^ *FOXP4* represses expression of *SOX2*,^32^ a known causal gene for microphthalmia/anophthalmia,^33^ and regulates TGF-β signaling.^34^ The variant has a CADD score of 33, a REVEL score of 0.843, and is predicted to be deleterious by SIFT and possibly damaging by PolyPhen. It is also exceedingly rare in the general population with a gnomAD (v4.1) allele frequency of 2.4 × 10^−5^ (including no homozygotes). Further, Sanger sequencing showed that *FOXP4* c.1433 A>G was absent in both unaffected relatives and present in all but one affected family member available for sampling (Fig. 1). Because the substitution sits near an intron–exon boundary and has a SpliceAI^35^ score of 0.59, we additionally sequenced cDNA derived from familial whole blood RNA samples. This demonstrated the presence of both the WT and missense transcripts without aberrant splicing (Supplementary Fig. S2), suggesting that the consequences of this variant likely manifest at the protein level.

**Figure 2.**
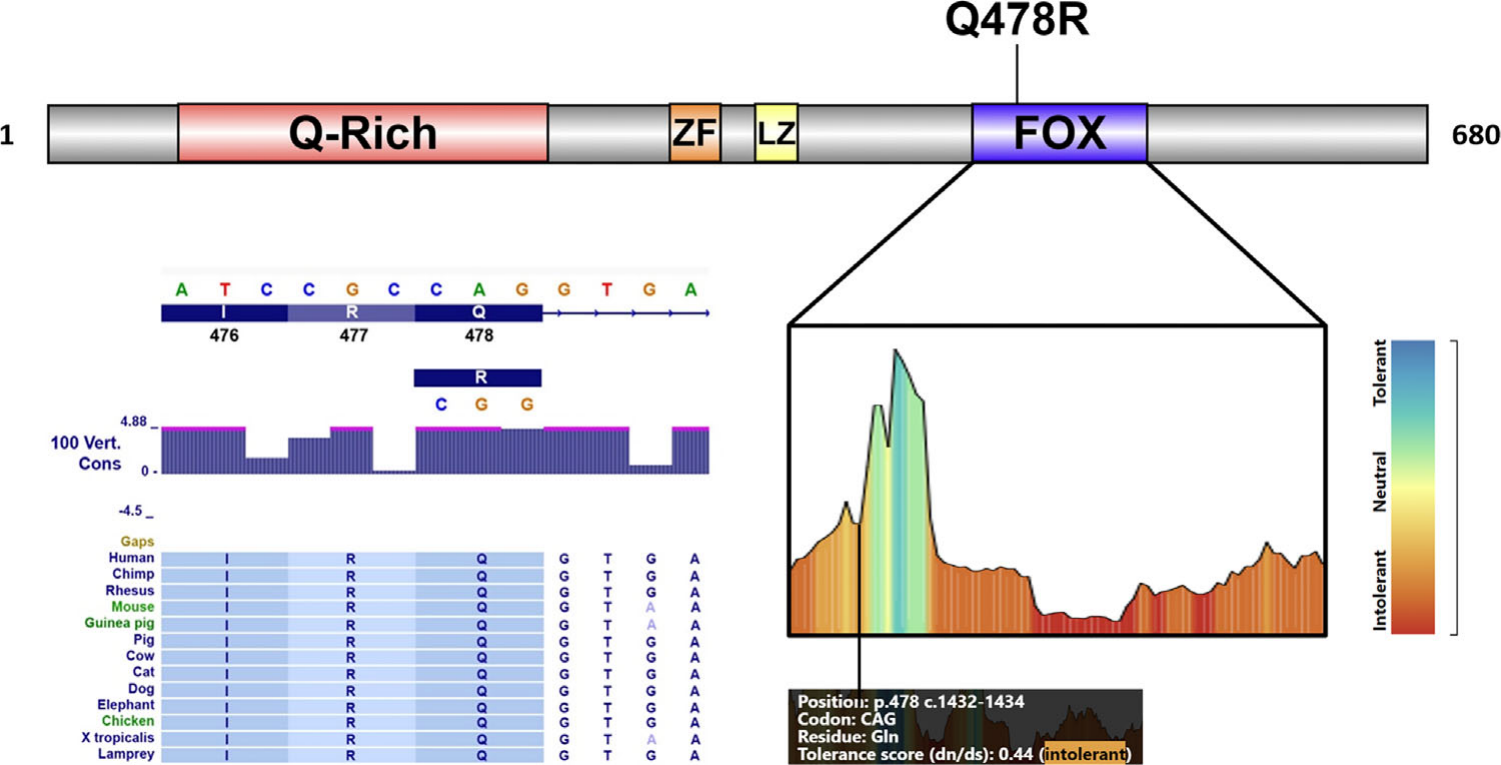
Structure of the FOXP4 protein,^22^ along with the tolerance landscape of the forkhead domain (amino acids 456–542)^22^^,^^37^ and the evolutionary conservation of the Arginine 478 residue.^36^ FOX, forkhead domain, LZ, leucine zipper; Q-rich, glutamine-rich; ZF, zinc finger.

Indeed, the mutation occurs in the DNA-binding forkhead domain at a residue that is both evolutionarily conserved^36^ and intolerant to variation, with a dn/ds score of 0.44^37^ (Fig. 2). The forkhead domain in FOXP4, as in other forkhead transcription factor family members, is generally intolerant to amino acid substitutions overall.^37^ Missense variants in this region were the most frequently reported cause of *FOXP4* loss of function in a cohort of pediatric patients with a dominant multisystem disorder.^22^ Features of this disorder include facial dysmorphisms and developmental delay, as well as ptosis and strabismus, although detailed ophthalmic examinations to ascertain for plateau iris, angle closure, or reduced axial lengths were not conducted at the time of the study and recontact was not possible (L. Snijders-Blok and S. Fisher, personal communication, 2021). None of the family members described in this report were notable for syndromic features upon initial assessment, but recontact was similarly infeasible, and thus the presence of more subtle systemic phenotypes cannot be excluded.

Ultimately, we identified no other rare variants in eye-expressed genes that both segregated among most family members and had suggestive in silico pathogenicity scores. This finding implicates *FOXP4* p.Q478R as a strong risk factor for the plateau iris, ACG, and short axial length phenotypes in this family.

### *FOXP4* Expression Patterns

**Figure 3.**
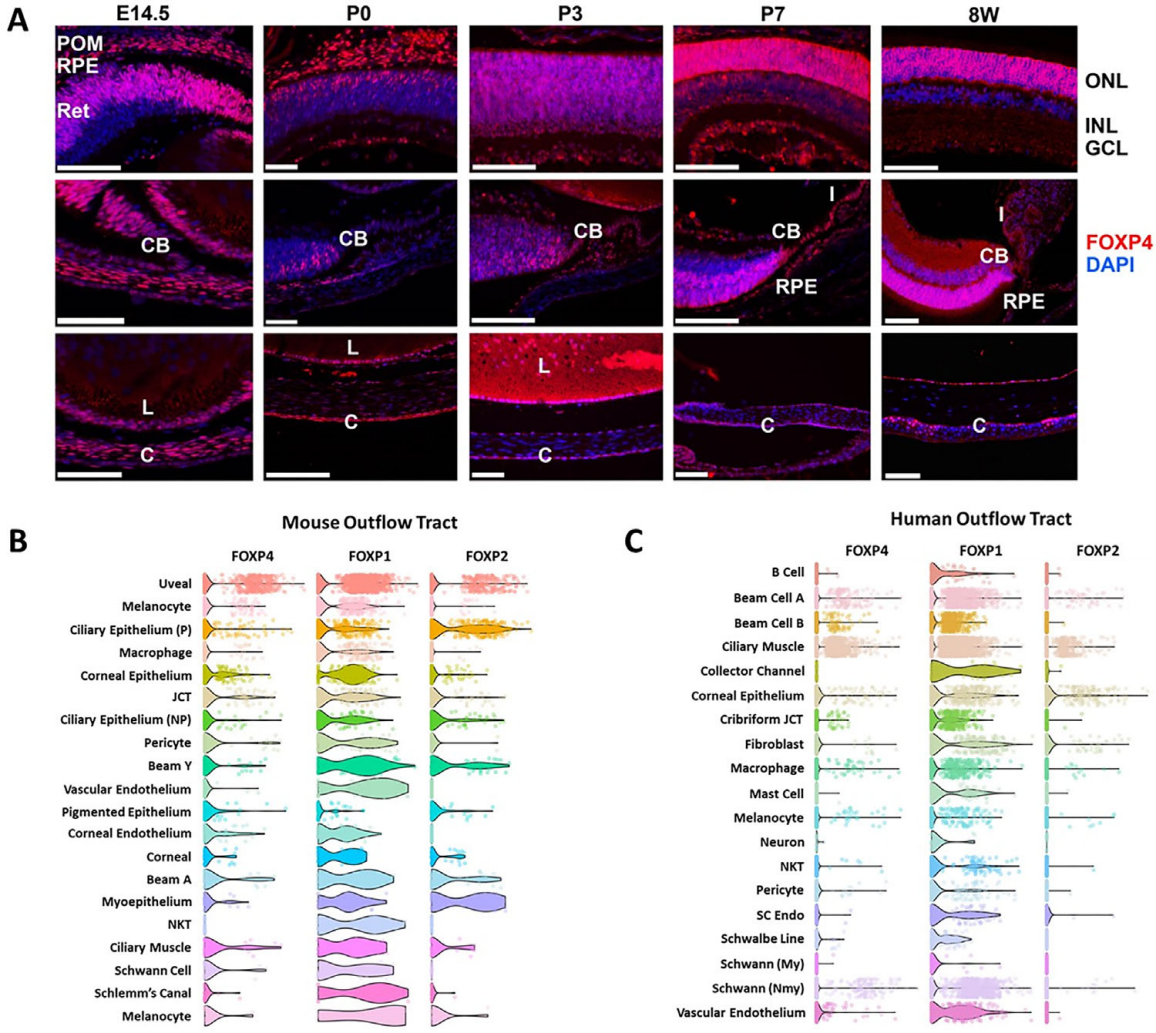
Spatiotemporal expression pattern of FOXP4 in developing mouse eyes from embryonic day 14.5 to 8 weeks (**A**). This is complemented by single-cell RNA sequencing from mature human (**B**) and mouse (**C**) eyes.^24^^,^^25^ C, cornea; CB, ciliary body; GCL, ganglion cell layer; I, iris; INL, inner nuclear layer; L, lens; ONL, outer nuclear layer; POM, periocular mesenchyme; Ret, retina. Scale bar, 25 µm.

To establish a potential role of FOXP4 in anterior segment development, we systematically evaluated its spatiotemporal expression patterns in mouse eyes using an antibody whose staining pattern has been validated via RNAscope in situ hybridization (Supplementary Fig. S3A). FOXP4 is observed consistently in the retina, RPE, lens/lens epithelium, and cornea/corneal epithelium throughout eye development and in mature mouse eyes. At E14.5, FOXP4 also shows high expression in the periocular mesenchyme, from which many anterior segment structures are derived.^38^ Additionally, FOXP4 is seen in the iris and ciliary body by P3. Reanalysis of single-cell sequencing data from adult humans and mice further supports high expression of FOXP4 and dimerization partners FOXP1 and FOXP2^39^ throughout the aqueous outflow pathway/anterior chamber, including the ciliary muscle, juxtacanalicular tissue, and Schlemm's canal (Figs. 3B, 3C).^24^^,^^25^ This finding is in agreement with a recent study showing that the high accessibility of FOXP1/FOXP2/FOXP4 binding motifs in murine trabecular meshwork correlates with the repression of target transcripts.^40^

### Functional Analyses

**Figure 4.**
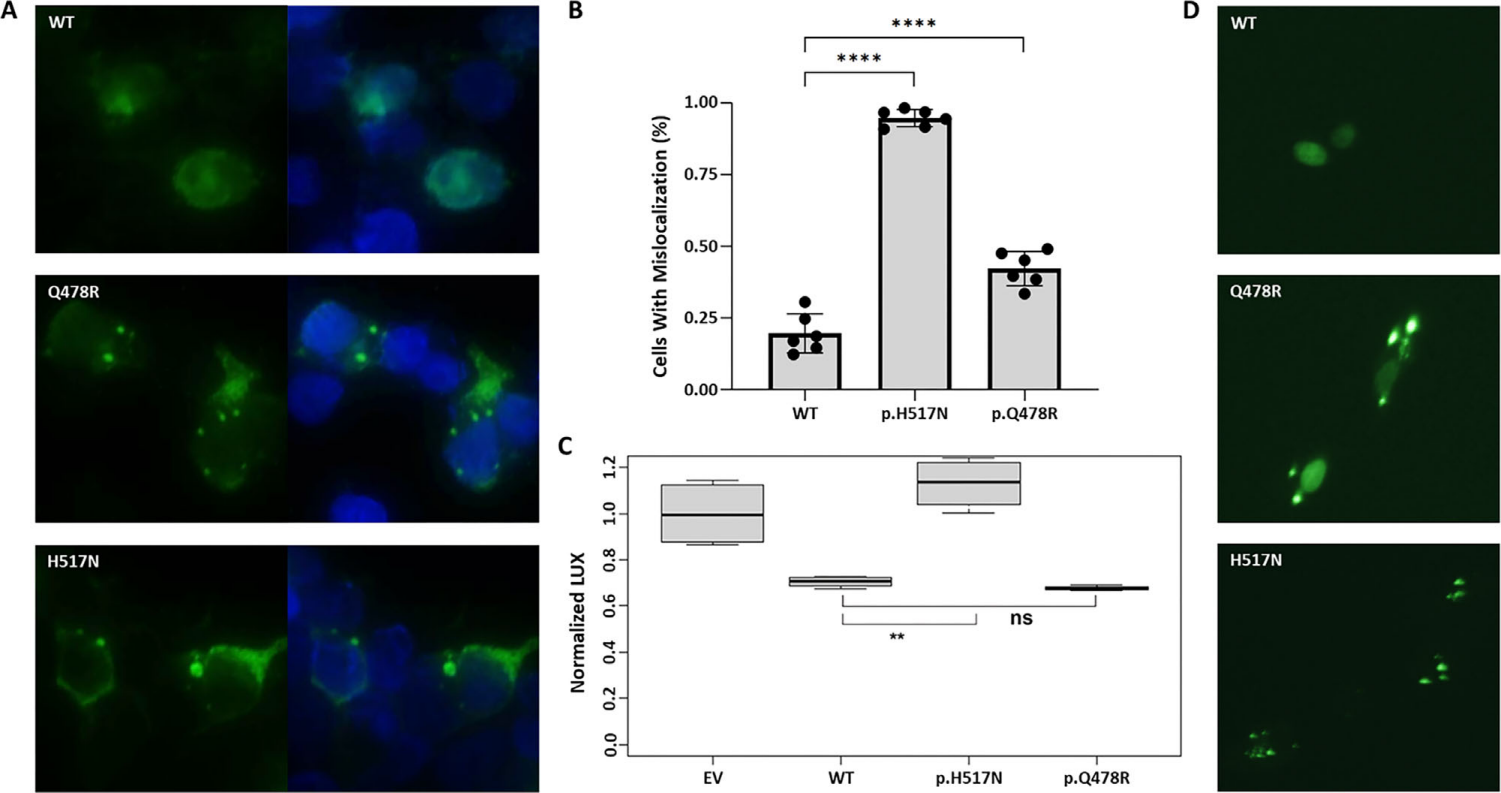
Representative images showing the localization of FOXP4 WT, Q478R, and H517N at an original magnification of ×40 in HEK-293T cells (**A**). In the same cell line, mislocalization of WT and variant proteins was quantified over six replicates (**B**) and luciferase assays conducted (**C**). Representative images showing the localization of FOXP4 WT, Q478R, and H517N at an original magnification of ×10 in ARPE-19 cells (**D**). *Green*, YFP-FOXP4; *blue*, DAPI; Normalized LUX, *Firefly/Renilla* luciferase expression ratio normalized to the empty vector control; EV, empty vector; ns, not significant;. **P < 0.01; ****P < 0.0001.

WT FOXP4 exhibits diffuse nuclear localization, while certain pathogenic FOXP4 mutations, particularly those within the forkhead domain, cause mislocalization to the cytosol.^22^ To determine the effects of our mutation on FOXP4 localization, HEK-293T cells were transfected with constructs expressing YFP-tagged FOXP4 WT, p.Q478R, or p.H517N, a known pathogenic variant^22^ (Figs. 4A, 4B; Supplementary Fig. S4). The WT protein was predominantly observed in the nucleus, with only 20 ± 7% of cells showing any additional cytoplasmic localization. In line with a previous report,^22^ 95 ± 3% of cells expressing the p.H517N mutant showed cytosolic localization, typically in the form of distinct aggregates with no accompanying nuclear signal. FOXP4 p.Q478R demonstrated characteristics of both WT and p.H517N FOXP4. We found that 42 ± 6% of cells expressing this construct showed cytosolic localization, which usually presented as either distinct cytosolic aggregates coupled with diffuse nuclear localization or diffuse localization throughout the cell. This phenotype was also prominently recapitulated in a more ocularly relevant RPE (ARPE-19) cell line (Fig. 4D; Supplementary Fig. S4).

To determine whether FOXP4 p.Q478R retained transcriptional activity, we evaluated its ability to repress the expression of a validated SPRX2-Luciferase reporter construct in HEK-293T cells.^22^ Unlike the p.H517N variant, FOXP4 p.Q478R was able to repress luciferase expression at levels comparable with WT at high transfection concentrations (Fig. 4C). These data, together with the previous experiment, suggest that FOXP4 p.Q478R is a hypomorph that retains repressive function but is localized improperly. Interestingly, when the luciferase assay was repeated at the much lower *FOXP4* transfection concentration used to detect localization differences, there was no significant change in luciferase expression between the WT and empty vector controls (Supplementary Fig. S5). This experiment may, therefore, lack an adequate dynamic range for the evaluation of hypomorphic alleles at biologically relevant expression levels.

### Cohort Screenings

To determine the prevalence of *FOXP4* mutations in cases of ACG, we first screened an in-house cohort of 37 patients diagnosed with plateau iris, ACG, and/or high hyperopia. This testing returned no variants likely to be deleterious.

We next reviewed a cohort of approximately 20,000 individuals with glaucoma and anterior segment conditions for whom variant calling files had been deposited in Genebass.^23^ These data revealed a nominally, though not genome-wide, significant enrichment of *FOXP4* loss-of-function (SKAT-O *P* = 1.45 × 10^−2^) and missense (SKAT-O *P* = 3.73 × 10^−2^) variants in patients diagnosed with disorders of the iris/ciliary body. We did not see a similar relationship between *FOXP4* variants and glaucoma despite prior GWAS data supporting a clear association.^19^ This likely due, in part, to limitations with the dataset (including the lack of an appropriate control population) in combination with the severity of FOXP4 loss-of-function phenotypes.^22^

### Protein Stability

**Figure 5.**
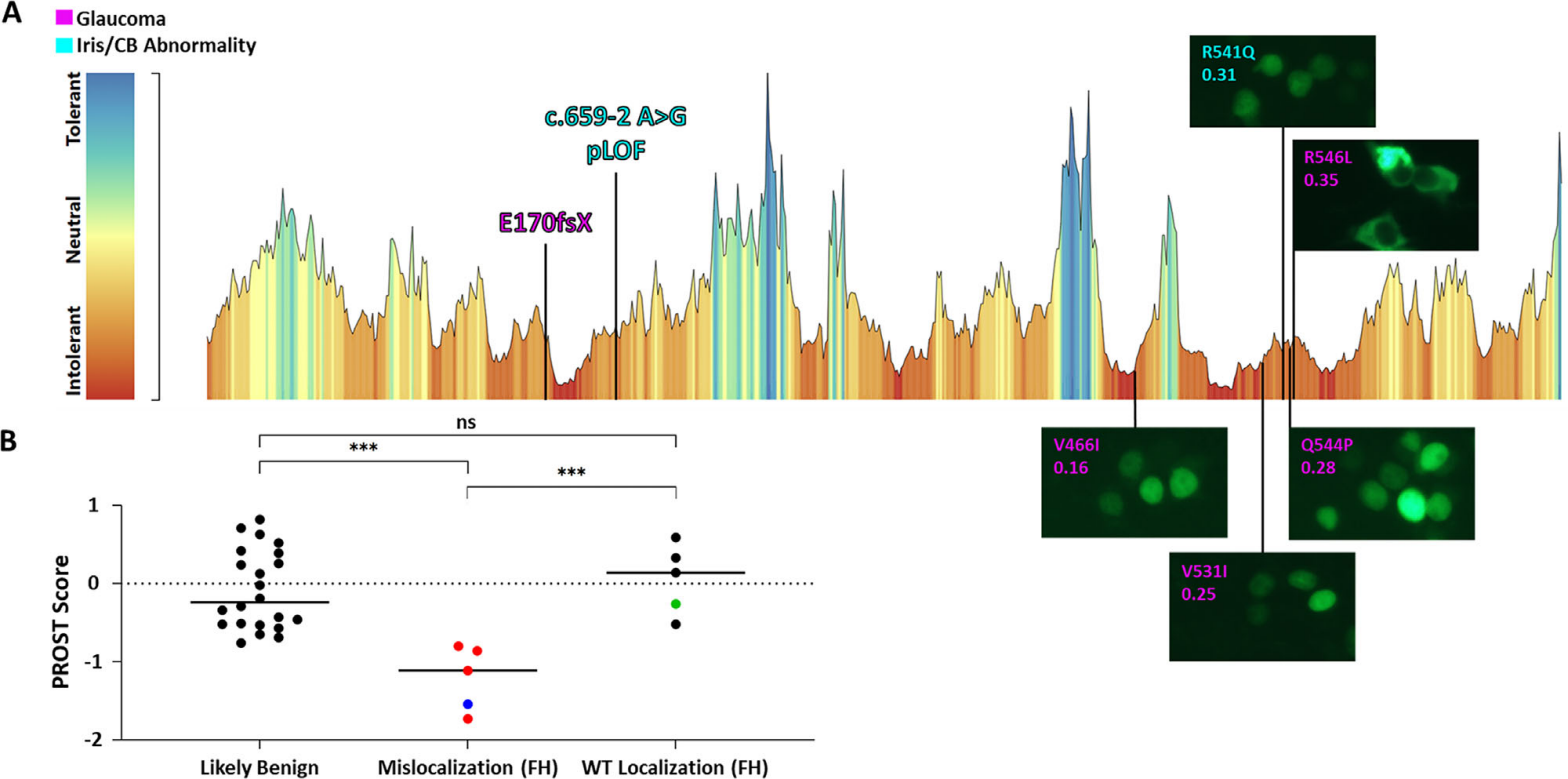
Predicted loss-of-function (pLOF) and forkhead variants found in the Genebass cohort of patients with glaucoma or disorders of the iris/ciliary body plotted along the FOXP4 tolerance landscape.^37^ Localization of the missense substitutions is visualized at an original magnification of ×20 and dn/ds scores for the residues at which they occur are noted (**A**). PROST scores were used to predict the stability of the mislocalizing forkhead variants as compared to likely benign variants and other forkhead variants that showed WT localization (**B**). *Red dots*, cytosolic mislocalization/aggregation; *Blue dots*, granular nuclear localization; *Green dots*, pathogenic variants that do not mislocalize. ***P < 0.001; ns, not significant; FH, forkhead.

To date, all *FOXP4* missense substitutions known to be pathogenic occur in and around the forkhead domain and often result in the protein localizing either to the cytosol or, in one case, the nucleus in a granular pattern.^22^ Such aberrant localization, particularly in the presence of aggregates, may be indicative of protein instability. To test this, we first parsed genomic data from Genebass participants diagnosed with iris/ciliary body disorders for forkhead proximal variants. We then did the same for all Genebass glaucoma patients because *FOXP4* is a known GWAS locus for OAG.^19^ Five mutations were ultimately identified, all of which occur at residues predicted to be intolerant to missense substitution^37^: p.V466I (c.1396G>A), p.V531I (c.1591G>A), p.R541Q (c.1622G>A), p.Q544P (c.1631A>C), and p.R546L (c.1637G>T) (Table 1; Fig. 5A). Although p.Q544P and p.R546L each appeared in only one Genebass participant, the exact allele frequencies in cases versus unaffected controls could not be determined for the remaining variants because we had to combine variant calling files from several nonindependent patient cohorts. Notably, this analysis also returned two variants predicted to result in complete loss of the forkhead domain if a stable protein were even produced: p.E170GfsTer145 (c.509_510del) and c.659-2 A>G (Splice AI = 0.96). These variants had gnomAD v4.1 allele frequencies of 6.2 × 10^−7^ and 1.2 × 10^−5^, respectively.

**Table 1.** Summary of the FOXP4 Forkhead Variants Found in Genebass Cohorts of Patients With Glaucoma or Disorders of the Iris/Ciliary Body

Substitution	PolyPhen	SIFT	gnomAD Allele Frequency	GeneBass Total Allele Count	CADD	REVEL	GERP
p.V466I	Deleterious low confidence	Possibly damaging	1.0E−05	3	24.5	0.48	4.1
p.V531I	Deleterious low confidence	Possibly damaging	1.9E−05	27	27.4	0.61	4.1
p.R541Q	Tolerated low confidence	Benign	4.0E−05	27	23.4	0.27	4.3
p.Q544P	Deleterious low confidence	Probably damaging	6.2E−07	1	28.8	0.87	4.3
p.R546L	Deleterious low confidence	Probably damaging	6.2E−07	1	32	0.92	4.3

GERP scores were added as a measure of conservation^68^ and gnomAD v4.1 allele frequencies given.

Next, to determine whether the identified forkhead substitutions resulted in protein mislocalization, we tested them in HEK-293T cells as before. FOXP4 p.V466I, p.V531I, p.R541Q, and p.Q544P displayed WT nuclear localization, whereas p.R546L showed primarily cytosolic localization, similar to p.H517N (Fig. 5A).

Finally, we used PROST, an AlphaFold2-informed in silico modeler that estimates the change in Gibb's free energy caused by missense mutations,^26^ to determine whether protein mislocalization correlated with predicted protein instability. We started by calculating PROST scores for all *FOXP4* missense variants on gnomAD v4.1 that were in greater than 10 people and classified as benign or tolerated by both PolyPhen and SIFT. Variants found in fewer than 10 people were also included if, in addition to meeting the PolyPhen and SIFT criteria, they had CADD scores of less than 13 and REVEL scores of less than 0.15. The average PROST score for this collection of 22 likely benign variants, which included no forkhead substitutions, was −0.08. We then calculated the average PROST score of all *FOXP4* substitutions experimentally validated as deleterious, which came to −1.05. Interestingly, the single deleterious variant with a score greater than −0.8 was p.A514T (−0.26), the only one that has a WT localization pattern^22^ (Table 2).

**Table 2.** Summary of In Silico Predictions and gnomAD v4.1 Allele Frequencies for Forkhead (FH) Variants That Have Undergone Functional Validation,^22^ as Well as Likely Benign Variants

Substitution	PolyPhen	SIFT	Allele Frequency	CADD	REVEL	PROST
Mislocalizing FH variant						
p.Q478R^*^	Deleterious	Possibly damaging	2.4E-05	33	0.84	−0.80
p.Y503C^*^	Deleterious low confidence	Probably damaging	6.2E-07	31	1.00	−1.11
p.H517N^*^	Deleterious	Probably damaging	0	25.4	0.96	−1.73
p.N518S^†^	Deleterious low confidence	Probably damaging	6.2E-07	27.8	0.93	−1.54
p.R546L^*^	Deleterious low confidence	Probably damaging	6.2E-07	32	0.92	−0.86
		Average:	5.2E-06	29.84	0.93	−1.21
WT localizing FH variant						
p.V466L	Deleterious low confidence	Possibly damaging	1.0E-05	24.5	0.48	0.33
p.A514T^‡^	Deleterious	Probably damaging	0	25.8	0.74	−0.26
p.V531I	Deleterious low confidence	Possibly damaging	1.9E-05	27.4	0.61	0.14
p.R541Q	Tolerated low confidence	Benign	4.0E-05	23.4	0.27	0.59
p.Q544P	Deleterious low confidence	Probably damaging	6.2E-07	28.8	0.87	−0.52
		Average:	1.4E-05	25.98	0.59	0.06
Likely benign						
p.G32S	Benign	Tolerated low confidence	4.9E-05	10.1	0.20	−0.76
p.G33R	Benign	Tolerated low confidence	3.9E-03	15.9	0.20	−0.53
p.T35I	Benign	Tolerated low confidence	2.7E-05	8.24	0.21	−0.46
p.T42K	Benign	Tolerated low confidence	2.9E-05	4.66	0.12	0.24
p.T42M	Benign	Tolerated low confidence	9.9E-06	10.3	0.14	0.13
p.T48M	Benign	Tolerated low confidence	7.4E-06	10.6	0.14	−0.19
p.T48K	Benign	Tolerated low confidence	6.8E-06	6.57	0.13	0.39
p.A59T	Benign	Tolerated low confidence	2.9E-05	16.8	0.17	−0.65
p.P166Q	Benign	Tolerated	6.8E-05	13.4	0.12	0.82
p.P166L	Benign	Tolerated	5.2E-05	18.4	0.05	−0.02
p.N196K	Benign	Tolerated	2.4E-05	18.9	0.13	−0.57
p.N208S	Benign	Tolerated	3.2E-05	4.19	0.06	0.42
p.V245I	Benign	Tolerated	4.3E-05	16.4	0.10	0.63
p.A264T	Benign	Tolerated	1.4E-05	10.3	0.01	0.26
p.A264V	Benign	Tolerated	7.6E-06	10.6	0.05	−0.43
p.P265S	Benign	Tolerated	1.8E-04	11	0.03	−0.34
p.L272F	Benign	Tolerated	1.2E-05	19.6	0.16	−0.52
p.G299S	Benign	Tolerated	1.9E-05	10.5	0.02	0.71
p.P386L	Benign	Tolerated	3.6E-04	10.3	0.06	−0.69
p.V398I	Benign	Tolerated	1.5E-04	13.5	0.16	0.52
p.A597T	Benign	Tolerated	5.7E-03	14.1	0.11	−0.29
p.S662L	Benign	Tolerated low confidence	7.2E-05	4.5	0.27	−0.51
		Average:	4.9E-04	11.77	0.12	−0.08

* Cytosolic mislocalization/aggregation.

† Granular nuclear localization.

‡ Pathogenic variants that do not mislocalize.

To control for the effects of domain constraint, which often influences in silico modeling, we further used PROST to evaluate forkhead variants relative to each other. Between our study and the one previous,^22^ 10 missense substitutions in this region have been examined for localization. We split these variants into two groups independent of their pathogenic status: those that mislocalize and those that do not. The average PROST score for the former was −1.21, whereas the latter came to only 0.06 (Table 2). As such, PROST was able to accurately differentiate between the two groups of forkhead variants (*P* = 8.6 × 10^−4^), as well as between forkhead variants that mislocalize and the likely benign variants curated above (*P* = 1.9 × 10^−4^), but not between likely benign variants and forkhead variants that do not mislocalize (*P* = 0.83). Indeed, there were no overlapping scores between mislocalizing variants and those in the other two categories (Fig. 5B; Table 2). In silico scores suggestive of protein instability thus tracked specifically with aberrant localization rather than variant domain or pathogenicity, indicating a possible mechanism by which this phenotype occurs.

## Discussion

During development, transcriptional cascades travel through the RPE to other ocular tissues—such as the periocular mesenchyme—and trigger growth factor expression/extracellular matrix remodeling. This process modulates many of the developmental risk factors for glaucoma, particularly eye size and anterior segment morphology.^41^^–^^47^ Forkhead box transcription factors are being increasingly recognized as key players in this process via their role in ocular pathologies, with mutations in individual forkhead genes responsible for a broad phenotypic spectrum of ASD/glaucoma based on type and severity (i.e., gain of function vs. loss of function).^17^^–^^19^^,^^40^ *FOXP4*, for instance, was found previously to be a significant GWAS locus for OAG.^19^ Here, we present evidence that variants in *FOXP4* are also associated with plateau iris and ACG.

Pooled whole exome sequencing of an Ashkenazi family with an autosomal dominant triad of plateau iris, ACG, and short axial lengths identified a single plausible candidate variant: *FOXP4* p.Q478R. This variant is absent in both definitively unaffected individuals and found in all but one of the affected family members with confirmed genotypes. Regarding the outlier, we note that plateau iris is a subjective clinical diagnosis that may be biased by family history. It is also exceedingly common in ACG patients (more than one-third),^48^ as is hyperopia (one-third to one-half).^49^^,^^50^ Thus, given the high prevalence of ACG as a specific diagnosis (approximately 2.3% in the adult Israeli population^51^) and its myriad of genetic and environmental causes,^3^ instances of phenocopy/locus heterogeneity featuring one or all characteristics of this triad are not necessarily surprising in some families. Indeed, the only previous report of a Mendelian plateau iris and ACG pedigree included multiple family members diagnosed with plateau iris despite not carrying the causal deletion in *SPATA13*.^10^ The skewed Mendelian ratio of Generation III (six of seven siblings affected) is further suggestive of multiple underlying risk factors in our case, though no additional candidate variants were identified after extensive screening.

Moreover, in silico modeling and functional studies both support that *FOXP4* p.Q478R is a hypomorphic allele, which may explain why the family does not exhibit the syndromic features associated with full loss-of-function variants.^22^ Although the mutant protein can effectively repress transcription when overexpressed, indicating that the substitution does not hinder DNA binding or dimerization, it is prone to forming cytosolic aggregates. This would be expected to decrease the amount of functional transcription factor available at biologically relevant expression levels.

Our cohort studies suggest that similar *FOXP4* variants are likely to be a rare cause of plateau iris and ACG overall, although it is interesting that *FOXP4* has a nominally significant association with iris/ciliary body disorders in Genebass. The fact that the same association was not observed with glaucoma may be due to several limitations with the database itself, given that variants in even a known disease gene like *OPTN*^52^ fall far short of genome-wide significance. In particular, the inability to enrich for specific disease presentations despite their disparate molecular etiologies, and the use of a control cohort in which nearly 60% of participants are actually under the typical age of disease onset,^53^ restrict the efficacy of rare variant burden testing. Such is especially true when considering genes linked to severe, dominant phenotypes that may make it difficult for adult-aged variant carriers to consent to genetic testing.

Despite these limitations, we were able to use Genebass to identify another glaucoma patient carrying a forkhead-proximal mutation (p.R546L) that results in cytosolic localization with some aggregate formation. Clinical details for this patient could not be obtained, but recent reports have suggested that *FOXP4* may also be associated with OAG.^19^ This is not unexpected as many genes have been linked to both subconditions, including *GLIS3* (another TGF-β–regulating transcription factor).^14^^,^^15^^,^^19^^,^^54^^–^^57^ In some cases, this may be caused by mutational spectrums. In other cases, however, examples of multiple members of the same family differentially developing ACG or OAG in response to the same mutation indicated that complex modifier effects may determine which endophenotypes arise from a specific molecular insult.^55^^,^^56^

For at least ACG, the pathogenic mechanism of *FOXP4* variants appears to be mislocalization/aggregation. There are multiple ways in which this may result in abnormal anterior segment morphology, especially since *FOXP4* is highly expressed in relevant tissue types—including the RPE, periocular mesenchyme, iris, and ciliary body—during development.^46^^,^^47^ The aggregates themselves may be cytotoxic. Additionally, the mislocalization may present a dosage problem wherein an insufficient amount of FOXP4 protein localizes to the nucleus to properly regulate target transcripts important for ocular development, such as *SOX2*.^32^

The exact cause of the aggregation shown here remains unclear, although forkhead substitutions that cause both full and partial mislocalization are well-documented in *FOXP1*, *FOXP2*, *FOXC1*, and *FOXC2*.^22^^,^^58^^–^^61^ Interestingly, although no nuclear localization sequence (NLS) has ever been characterized in *FOXP4*, *FOXC1* is known to have two such sequences flanking the forkhead domain.^60^ This suggests that forkhead-proximal variants may result in aberrant localization by either direct substitution at a necessary residue in an NLS or by causing local instability that interferes with the function of an NLS. They may also destabilize the protein as a whole. Indeed, PROST free energy scores were indicative of at least some degree of instability among mislocalizing variant proteins. This analysis additionally revealed that PROST was able to differentiate mislocalizing forkhead substitutions from not only likely benign variants, but nonmislocalizing forkhead mutations with a high degree of specificity. Thus, PROST is a valuable new tool for predicting the consequences of amino acid substitutions.

Ultimately, these data support an important role for *FOXP4* in anterior segment development and suggest that *FOXP4* variants may be highly penetrant risk factors for plateau iris/ACG. It also adds to the growing list of genes associated with both multisystem disorders and isolated glaucoma depending on dosage and/or modifier effects, with other examples including *GLIS3*, *LMX1B*, *MEIS2*, *MYRF*, and *CDH11*.^14^^,^^15^^,^^19^^,^^62^^–^^67^ *FOXP4* syndromic patients should, therefore, receive regular ophthalmic care to help prevent visual field loss in cases where glaucoma treatment is appropriate, although these patients may be difficult to examine in childhood. The broad developmental expression of *FOXP4* and its role in anterior segment disease also warrant further studies exploring the links between other FOXP transcription factors, such as dimerization partners *FOXP1* and *FOXP2*, in glaucoma and ASD.

## Supplementary Material

Supplement 1
