# Supplementary material for: FOXP4 Variants Are Associated With Plateau Iris and Angle Closure Glaucoma
Source: Invest Ophthalmol Vis Sci. 2025 Jul 10;66(9):23. doi: 10.1167/iovs.66.9.23 (PMC12255186; doi:10.1167/iovs.66.9.23)
Supplement: Supplement 1 [file iovs-66-9-23_s001.pdf]

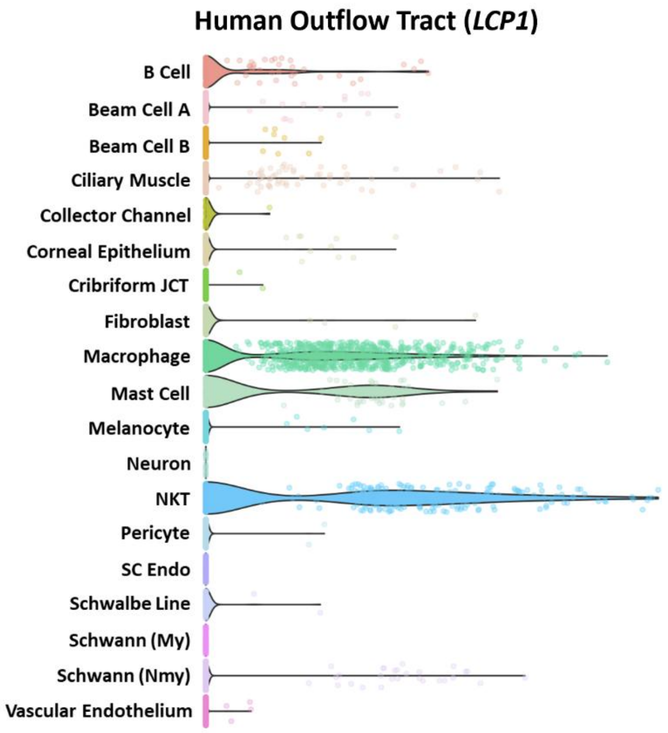

Figure S1: Expression pattern of *LCP1* in the human outflow tract[24, 25].

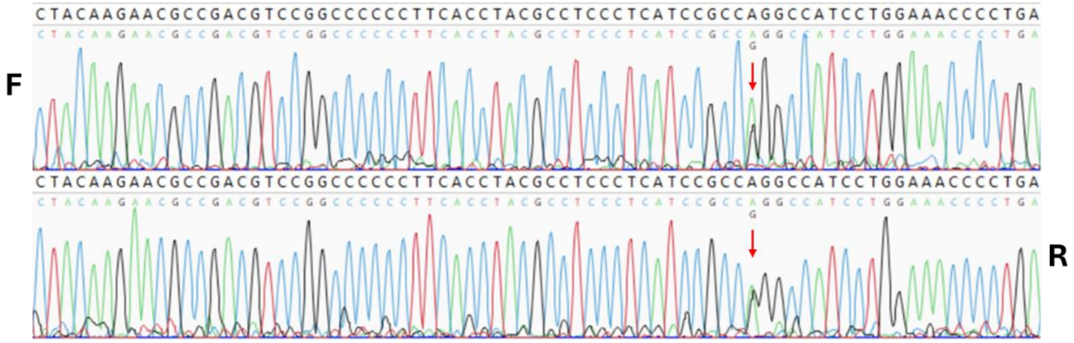

Figure S2: Sanger sequencing of cDNA from a patient carrying the p.Q478R variant in both the forward (F) and reverse (R) directions.

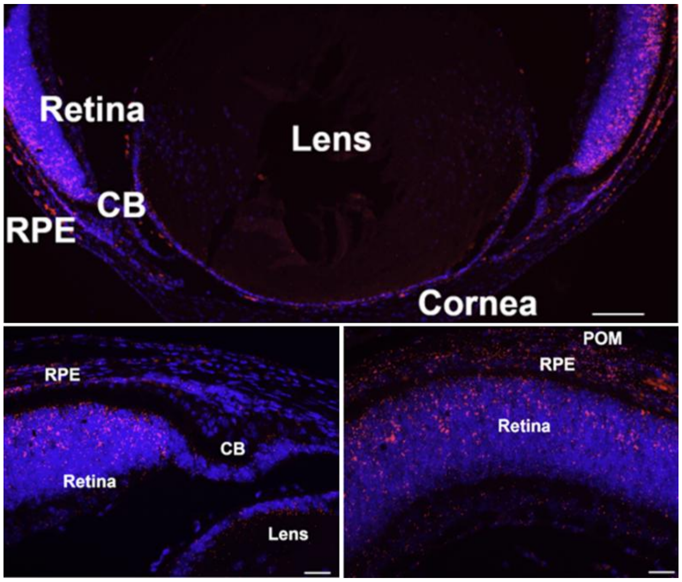

Figure S3: RNAscope *in situ* hybridization in a P0 mouse against *FOXP4* (red). Nuclei are counter-stained in blue. CB, ciliary body; RPE, retinal pigment epithelium, POM, periocular mesenchyme.

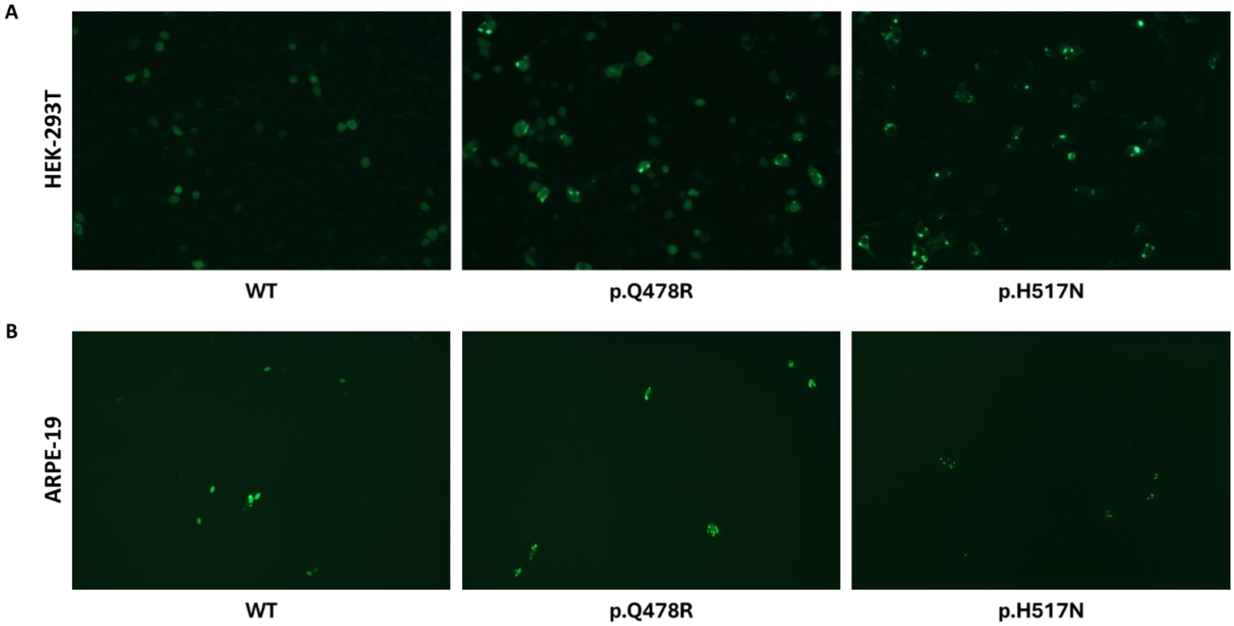

Figure S4: Representative images of HEK-293T (A) and ARPE-19 (B) cell plates transfected with *FOXP4* WT, p.Q478R, or p.H517N. Magnification objectives are 20x and 10x respectively.

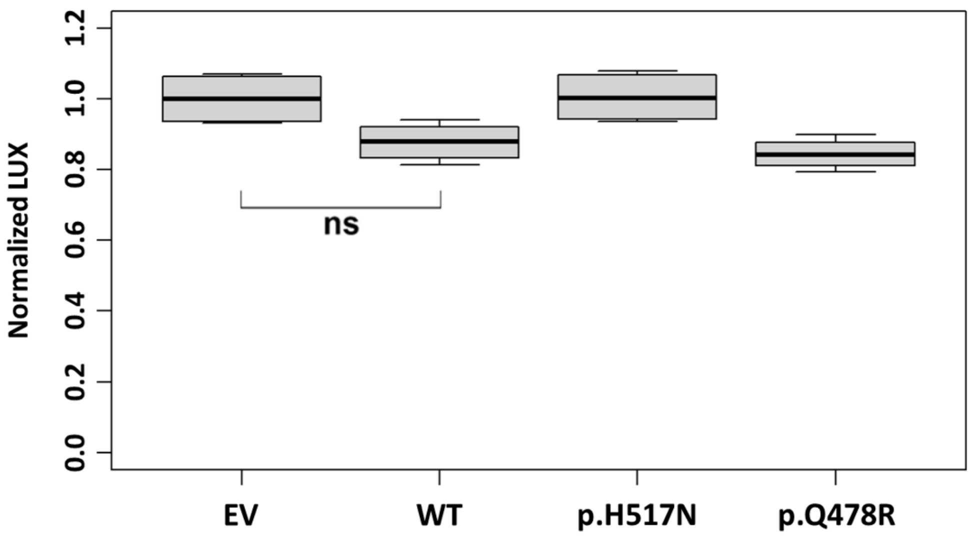

Figure S5: Luciferase assay conducted with only 20ng of *FOXP4* plasmid per condition. EV, empty vector; ns, not significant.

Primer Name	Sequence	Purpose	Annealing Temp
FOXP4 Q478R Forward	GAGCATGGATGCTGAGGCAA	PCR/Sanger	61
FOXP4 Q478R Reverse	GGGACAGCTCCATTGCTTGG	PCR/Sanger	61
LCP1 K534T Forward	ATGGTGTCCAGACTGTCTTCT	PCR/Sanger	58
LCP1 K534T Reverse	GAGGGGGAGCCTCTAATACCT	PCR/Sanger	58
FOXP4 Splice Check Forward	CTCATTCTCCAAGGTGACCG	RT-PCR/Sanger	58
FOXP4 Splice Check Reverse	CTCCGCTTCTGATACTCCCG	RT-PCR/Sanger	58
FOXP4 Q478R Mut Forward	CCCTCATCCGCCGGGCCATCCTGGA	Mutagenesis	60
FOXP4 Q478R Mut Reverse	TCCAGGATGGCCCGGCGGATGAGGG	Mutagenesis	60
FOXP4 V466I Forward	ATGAGTTCTACAAGAACGCCGACATCCGGCCC	Mutagenesis	60
FOXP4 V466I Reverse	GGCGTAGGTGAAGGGGGGCCGGATGTCGGCGT	Mutagenesis	60
FOXP4 V531I Forward	AGTGCTTCGTCCGCGTGGAGAACATCAAGGGT	Mutagenesis	56
FOXP4 V531I Reverse	CACAGTCCACACGGCACCCTTGATGTTCTCCA	Mutagenesis	56
FOXP4 R541Q Forward	TGCCGTGTGGACTGTGGACGAGCAGGAGTATC	Mutagenesis	60
FOXP4 R541Q Reverse	GCGGTCTCCGCTTCTGATACTCCTGCTCGTCC	Mutagenesis	60
FOXP4 Q544P Forward	GACTGTGGACGAGCGGGAGTATCCGAAGCGGA	Mutagenesis	60
FOXP4 Q544P Reverse	TCATCTTTGGCGGTCTCCGCTTCGGATACTCC	Mutagenesis	60
FOXP4 R546L Forward	GGACGAGCGGGAGTATCAGAAGCTGAGACCGC	Mutagenesis	56
FOXP4 R546L Reverse	TCCCTGTCATCTTTGGCGGTCTCAGCTTCTGA	Mutagenesis	56

Table S1: Primers used for PCR/Sanger sequencing and mutagenesis.

Accession	Generation/ Birth Order	Age at Exam	SE Refractive Error (D)		Axial Length (mm)		Avg Keratometry (D)		Gonio (Shaffer)		Iris Configuration	ACG Status	Lens Exam	
			OD	OS	OD	OS	OD	OS	OD	OS			OD	OS
N/A	II-4	70	7.5	8.75	N/A	N/A	N/A	N/A	slit	slit	Unknown	A	2+NS	2+ NS
G02272	III-1	67	3.5	3	21.27	21.46	43.75	44.25	N/A	N/A	Plateau	A	tr NS	tr NS
G02294	III-2	66	-1.5	N/A	23.11	22.86	45	45	4	4	Normal	U	tr NS 1+ CC	tr NS 1+ CC
G02342	III-4	61	2.75	4	20.8	20.49	45.25	45.5	slit	slit	Plateau	A	1+ NS	1 +NS
G02271	III-5	58	0.5	1.25	21.99	21.73	45.25	44.75	2-3	2-3	Plateau	U	1+ NS	1+ NS
G02341	III-6	55	1	2	21.62	21.07	44	44	slit	slit	Plateau	A	Clear	Clear
G02280	III-7	53	5	2.25	20.69	21.54	42.5	43	slit	slit	Plateau	A	1+ NS	1+ NS
G02339	IV-1	43	0.25	-5.5	25.38	26.19	40	40	IV	IV	Normal	U	PCIOL	1+ NS, 3+ PSC

Table S2: A summary of clinical features for individuals within the pedigree. SE, spherical equivalent; N/A, not available; ACG, angle closure glaucoma; A, affected; U, unaffected; NS, nuclear sclerosis; CC, cortical cataract; PSC, posterior subcapsular cataract.
